# Ploidy is associated with multielemental reorganization across soft and mineralized body compartments

**DOI:** 10.1098/rsbl.2026.0316

**Published:** 2026-10-14

**Authors:** Punidan Jeyasingh, Marissa Roseman, Josh Bliss, Yetkin Ipek, Maurine Neiman

**Affiliations:** ^1^ Department of Biology, Oklahoma State University, Stillwater, OK, USA; ^2^ Department of Biology, University of Iowa, Iowa City, IA, USA

**Keywords:** compositional data analysis, ecological stoichiometry, ionomics, genome size, polyploidy

## Abstract

Polyploidy alters nuclear DNA content, gene dosage and cellular organization, but its consequences for organismal chemistry remain poorly resolved, particularly in animals. Because nucleic acids and associated cellular machinery require coordinated allocation of multiple elements, ploidy-associated differences may extend beyond the well-established consequences for body phosphorus content to broader multielemental organization. We tested this hypothesis in the New Zealand mudsnail (*Potamopyrgus antipodarum*), which features within-population ploidy polymorphism, and exhibits strong compartmentalization between metabolically active soft tissue and mineralized shell. We quantified multielemental composition of soft tissue and shell and analysed allocation using additive log-ratios anchored to the measured elemental pool. Most multielemental variance was harboured in tissue versus shell, confirming distinct ionomic regimes for these two fundamental components of the molluscan body. Across both soft tissue and shell, ploidy was associated with significant redistribution of relative elemental allocation despite weak single-element effects. Ploidy-associated imbalances differed between shell and soft tissue, consistent with elemental physiology in inert and active tissues. Together, these results are consistent with a scenario where ploidy level is associated with subtle but coordinated redistribution of elemental allocation across biological compartments.

## 1. Introduction

Total nuclear genomic DNA content varies widely within and among species and is associated with cell size, genome size, gene content, gene dosage, ploidy level and physiological scaling [1–6]. Polyploidy is a form of nuclear genomic DNA content variation common across the evolutionary history of life that generates discrete, heritable differences in nuclear DNA content that can reshape organismal performance and life-history traits [5,7]. Despite extensive study, however, the mechanisms linking genome architecture to organismal phenotype remain incompletely resolved, particularly in animals, where polyploidy is less common and often context dependent [5,8].

One approach frames genome DNA content from a material perspective. Because DNA is phosphorus rich, variation in nuclear DNA content has been proposed to impose measurable phosphorus costs at cellular and organismal levels [9]. Under phosphorus limitation, selection may favour reduced genome DNA content if P allocated to DNA competes with P required for RNA and growth machinery [9]. Empirical work in both animals and plants shows that ploidy can influence performance under P limitation and, in some cases, bulk tissue %P [10,11]. However, broader comparative analyses indicate that relationships between genome size and tissue N or P concentrations are often weak or context dependent [12,13], suggesting that genome DNA content effects may be mediated through allocation trade-offs and physiological fluxes rather than simple proportional increases in bulk P content. In particular, nucleic acids operate within a broader biochemical network involving metal-dependent enzymes, ionic regulation and biomineralization processes [14,15]. If ploidy alters cellular organization, transcriptional demand or transport architecture, its effects may propagate beyond phosphorus alone and emerge as coordinated multielemental shifts. From this perspective, organisms can be viewed as dynamic material systems in which elemental composition reflects the integrated outcome of uptake, transport, storage, transformation and loss. Consequently, ploidy-associated differences need not be expressed through phosphorus alone but may emerge as coordinated shifts in the relative allocation of multiple elements.

The ionome, defined as the relative abundance of multiple chemical elements within a biological system, provides a natural phenotype for capturing such multielemental organization [16,17]. Because elemental composition is compositional, changes in one element necessarily imply compensatory shifts in others. Ecological stoichiometry formalizes this mass-balance logic and emphasizes that elemental allocation reflects the combined effects of physiological regulation and environmental supply [18]. Analyses that treat elements independently risk obscuring coordinated allocation patterns that emerge from these constraints. Genome DNA content variation should therefore be expected to reorganize elemental allocation across the organism rather than simply increase demand for any single element.

Compositional data analysis provides an appropriate statistical framework for such systems by accounting for closure and enabling inference on relative allocation among elements [19,20]. When total organismal chemistry is incompletely observed, the sum of measured elements can be treated as a filling value defining the observed compositional system and making explicit the unmeasured remainder. This approach permits robust comparison of multielemental allocation even when absolute mass or total chemistry are partially unknown, a common limitation in organismal ionomic datasets.

What, then, should genome DNA content variation affect within a multielemental biological system? A common expectation is that effects should be most evident in metabolically active tissues, where changes in transcriptional demand, cell size and biosynthetic scaling are expressed directly [2,3,5,21]. These processes rely heavily on coordinated elemental fluxes, particularly of phosphorus-rich nucleic acids and metal-dependent enzymes [18,22], suggesting that genome DNA content effects might be detectable as shifts in elemental allocation within soft tissues.

This possibility is complicated by the reality that metabolically active tissues are also subject to strong homeostatic regulation. Transport, storage and turnover mechanisms can buffer elemental composition against environmental variability and underlying genomic differences, potentially dampening DNA content contrasts at steady state [17,23,24]. An alternative possibility is that genome DNA content effects are expressed more clearly in metabolically inert or slowly turning-over tissues. Mineralized structures integrate elemental deposition over longer developmental timescales and may therefore preserve subtle persistent allocation biases that are buffered in actively regulated soft tissues [25]. Thus, tissues may differ not only in composition but also in the temporal scales over which elemental allocation is integrated.

These contrasting expectations motivate a simple question: are nuclear DNA content effects on elemental composition expressed primarily through short-term metabolic regulation or through long-term integration in mineralized tissue? Addressing this question requires a system in which genome DNA content variation is discrete, tissues differ sharply in metabolic activity, and multielemental composition can be quantified.

The New Zealand freshwater snail *Potamopyrgus antipodarum* provides such a system. Natural populations harbour diploid, triploid and tetraploid lineages [26] and exhibit pronounced compartmentalization between metabolically active body tissue and a mineralized shell. Here, we quantify ionomes of body tissue and shell and analyse composition using log-ratio methods that explicitly accommodate compositional constraints [19,20]. By focusing on coordinated shifts in multielemental allocation rather than isolated elemental changes, we test whether ploidy reshapes elemental composition more strongly in metabolically active tissue or in mineralized structure, thereby characterizing associations between ploidy level and ionomes.

## 2. Material and methods

We analysed lab-reared isofemale lineages of *P. antipodarum* established from two South Island, New Zealand lakes (Mapourika and Selfe) previously established as harbouring distinct assemblages of *P. antipodarum* [27], and thus allowing for replication of ploidy comparisons across two natural populations. Diploid (2n) and triploid (3n) lineages were sampled across three ontogenetic stages. For each individual, metabolically active soft tissue and mineralized shell were dissected and analysed separately (*n* = 208 tissue samples total). Ploidy of a representative individual for each lineage was characterized by flow cytometry following established protocols [26,28].

Body tissue and shell were acid-digested and analysed using inductively coupled plasma–optical emission spectroscopy (ICP–OES) to quantify a multielement panel. Because ICP–OES is optimized for metals and selected nonmetals but not for bulk C, H, N or O quantification, the present analysis focuses on the measured elemental pool rather than total organismal stoichiometry. Although bulk CHON elements dominate organismal biomass, they often exhibit relatively limited variation among closely related genotypes or evolutionary treatments compared with ionomic fractions involved in regulation, transport, catalysis and biomineralization (e.g. [29]). The present analysis therefore targets the multielemental component of organismal chemistry most probably to capture subtle compositional reorganization associated with ploidy. Concentrations are reported in solution units. Because sample mass was unavailable for a subset of samples, analyses focused on relative elemental allocation. Note that body tissue and shell were measured from the same snail, representing paired compartments rather than independent individuals. The primary analyses treated body part as a factor of interest because compartment-specific composition was central to the study. To assess robustness to the paired design, we took the mean of body and shell measurements within individuals and repeated the multivariate analyses using one observation per snail.

We defined a filling value (Fv) as the sum of 13 consistently detected elements and expressed elemental composition using a version of additive log-ratios

$$ALR_{i}=ln\left(x_{i}\;/\;Fv\right)$$

The primary inferential analysis was a fully factorial MANOVA including developmental stage, body part, lake of origin, ploidy and the body part × ploidy interaction. The principal inference regarding ploidy is based on the ploidy term from this full model. Box 1 summarizes the results of the full MANOVA, whereas table 1 presents a reduced MANOVA including only body part, ploidy and their interaction to facilitate interpretation of these focal biological effects. Significance of model terms was evaluated using Wilks's lambda multivariate effect tests. Body tissue and shell-specific linear models were used to estimate ploidy-associated elemental imbalances. Detailed rearing, sampling, processing and analytical protocols are provided in the supplementary material.

**Table 1.** Multivariate test statistics for body part and ploidy.

effect	Wilks's *λ*	F	num df	den df	*p*-value
reduced model	0.2245	10.22	36	559.15	< 0.0001
body part	0.2844	45.43	12	189	< 0.0001
ploidy	0.1229	1.94	12	189	0.0325
body part × ploidy	0.0209	0.33	12	189	0.9832

*Note:* Multivariate tests were conducted on additive log-ratios (ALRs; ln(element | Fv)) using MANOVA. Wilks's lambda (*λ*), approximate F-statistics, numerator and denominator degrees of freedom (df), sample size (n) and associated *p*-values are reported. Analyses were conducted on 13 ALRs corresponding to elements with ≤5% missing values. Results are shown for the reduced MANOVA including body part, ploidy and their interaction. The primary inference regarding ploidy is based on the full MANOVA summarized in box 1, whereas this reduced model is presented to simplify interpretation of the focal biological effects.

**Box 1 Hierarchy of biological variation in *P. antipodarum ionomes***

Ionomic composition reflects multiple nested sources of variation operating at distinct biological scales. The statistics reported below are derived from the primary MANOVA including developmental stage, body part, lake of origin, ploidy and the body part × ploidy interaction (*n* = 204 tissue samples; 13 additive log-ratios). Because the body part × ploidy interaction was not statistically significant, the hierarchy below emphasizes the main effects structuring ionomic variation. Table 1 presents a reduced MANOVA focused on the primary biological comparison between body part and ploidy.

— *Developmental stage:* Developmental stage exerted the strongest multivariate influence on ionomic composition (MANOVA, F_12_,_188_ = 92.89, *p* < 0.0001). This large effect reflects ontogenetic changes in traits and associated elemental signatures (e.g. [30]), and is consistent with other data demonstrating distinct body composition in juvenile and adult *P. antipodarum* [31]. Stage was included to prevent confounding ploidy with ontogenetic scaling.
— *Body compartment:* Body part (soft tissue vs. shell) was also a dominant determinant of elemental allocation (F_12_,_188_ = 45.45, *p* < 0.0001). This strong partition reflects fundamental differences in turnover rate, integration timescale and biomineralization processes. Tissue establishes the primary structural background against which other effects are expressed.
— *Lake of origin:* Lake contributed a significant but smaller multivariate effect (F_12_,_188_ = 7.17, *p* < 0.0001), consistent with differences in environmental elemental supply previously demonstrated for the New Zealand lakes that harbour *P. antipodarum* [32]. Because only two lakes were sampled, lake was treated as a fixed effect representing background geochemical context rather than as a focal ecological variable. Consequently, the present study does not estimate population-level lake effects.
— *Ploidy level:* Conditional on body part, developmental stage and lake, ploidy retained a statistically significant multivariate effect (F_12_,_188_ = 2.30, *p* = 0.0093). Although smaller in magnitude than other sources of ionomic variation, this effect emerged as coordinated redistribution across elements in ALR space and was rarely detectable at the single-element level. The persistence of this signal after conditioning on strong ontogenetic and structural partitioning indicates that ploidy is associated with subtle but coherent shifts in relative elemental allocation.

### (a) Sensitivity analyses

Because body and shell samples were obtained from the same individual and observations were distributed among multiple isofemale lineages, we conducted additional robustness analyses. First, to evaluate the paired sampling structure, we repeated the multivariate analysis using one observation per individual obtained by averaging body and shell ALRs. The ploidy effect remained significant (Wilks's *λ* = 0.759, *F* = 2.095, *p* = 0.022), indicating that the association between ploidy and ionomic composition was not dependent on treating body and shell measurements as independent observations. Second, to evaluate whether the ploidy-associated signal depended on unrestricted exchange of observations among lineages, we conducted a lineage-blocked permutation analysis in which permutations were restricted within isofemale lineages. The ploidy-associated signal remained significant under this analysis (PERMANOVA, *F* = 1.64, *p* = 0.001). These sensitivity analyses indicate that the observed association between ploidy and ionomic composition is not an artefact of paired tissue sampling or unrestricted permutation among lineages.

### (b) Boundary of inference

This study isolates ploidy as the primary axis of interest while statistically conditioning on body part, developmental stage and lake of origin. Developmental stage and body compartment explain the majority of multielemental variance, as expected for growth and compartmental partitioning. By contrast, the ploidy effect is smaller but remains detectable after accounting for these stronger sources of variation. Because diploid and triploid *P. antipodarum* also differ in reproductive mode, the present data support associations between ploidy and multielemental organization rather than direct causal attribution to DNA content alone. Furthermore, only two lakes and a finite set of isofemale lineages were sampled. Accordingly, inference is restricted to the sampled lake-derived lineages, while the broader relevance of the results derives from the generality of genome duplication as a recurring feature of eukaryotic evolution.

Furthermore, we do not necessarily expect all lineages sharing the same ploidy level to converge on identical ionomic states. If ploidy acts as a material perturbation affecting cellular organization, transport, biomineralization and elemental allocation, distinct lineages may accommodate that perturbation through different physiological and evolutionary trajectories. Consequently, lineage-level heterogeneity is biologically expected rather than simply statistical noise. For this reason, we interpret ploidy as a source of multielemental reorganization expressed against a background of lineage-specific variation rather than as a determinant of a single invariant ionomic phenotype.

## 3. Results

### (a) Ionomic quantification

Of the 25 elements measured (see supplementary material), 13 (B, Ca, Cu, Fe, K, Li, Mg, Mn, Na, P, S, Si, Zn) exhibited ≤5% missing values and were retained as a core ionomic panel for multivariate analyses. Twelve elements (Al, As, Ba, Cd, Co, Cr, Mo, Ni, Pb, Se, Sr, V) with high (more than 25%) missingness (i.e. below instrument LOD or true absence) were excluded from multivariate inference to avoid uneven sample representation and distortion of compositional covariance structure. When analysed together, ionomic data showed hierarchical structuring, with ploidy remaining a consistent effect (box 1). Although ploidy-associated effects were substantially weaker than those of developmental stage or body part, they remained statistically detectable after conditioning on these major sources of variation. Throughout the article, inference regarding the association between ploidy and multielemental organization is based on the ploidy term from this full MANOVA (box 1), whereas table 1 provides a simplified presentation of the focal body part and ploidy effects.

### (b) Body part defines distinct regimes of elemental allocation

Elemental allocation differed strongly between metabolically active soft tissue and mineralized shell (figure 1). In a reduced multivariate model including body part, ploidy and their interaction, body part explained a large proportion of multielemental variation in ALR space (table 1). This effect was independent of ploidy and indicates that soft tissue and shell occupy distinct ionomic regimes defined by relative elemental allocation rather than absolute concentrations.

**Figure 1.**
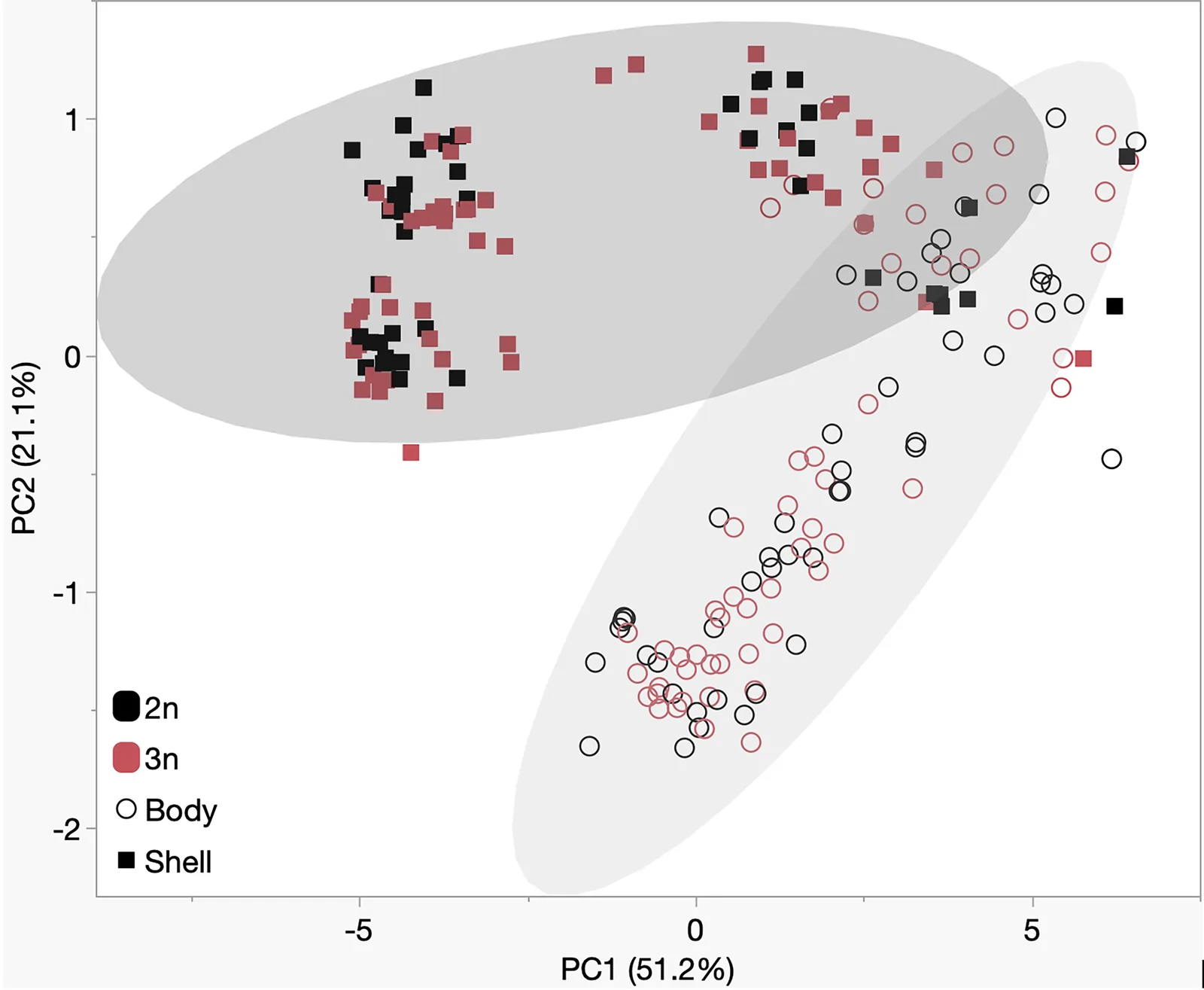
Body part defines distinct regimes of elemental allocation. Principal component analysis (PCA) of additive log-ratios (ALRs; ln(element | Fv)) for metabolically active soft tissue (circles) and mineralized shell (squares) from diploid (2n, black) and triploid (3n, red) P. antipodarum. Each point represents an individual sample. Shaded ellipses illustrate the overall distribution of body and shell samples in multivariate compositional space. Separation along the primary ordination axes indicates differences in relative elemental allocation between soft tissue and shell. Tissue type explained a large proportion of multielemental variation in ALR composition (MANOVA, Wilks's *λ* = 0.431, *F* = 20.87, *p* < 0.0001).

### (c) Ploidy reorganizes relative elemental allocation

Across tissues, ploidy was associated with significant multielemental differences in ALRs, suggesting that genome DNA content variation reorganizes relative elemental allocation within the measured elemental pool (table 1). Although weaker than the body part effect, this result demonstrates a detectable, system-wide influence of nuclear DNA content on elemental allocation conditional on Fv. The interaction between body part and ploidy was not supported (table 1), indicating that ploidy-associated ionomic reorganization is broadly consistent across metabolically active and mineralized tissues at the multivariate level.

Ploidy-associated elemental imbalances were further resolved at the level of individual elements using tissue-specific linear models in ALR space (figure 2). When expressed as ln(element | Fv), ploidy effects were centred on the diploid (2n) reference state, allowing direct interpretation of positive and negative departures in relative elemental allocation. In both tissues, ploidy effects were heterogeneous across elements, indicating selective reorganization of elemental fractions rather than uniform scaling of the ionome.

**Figure 2.**
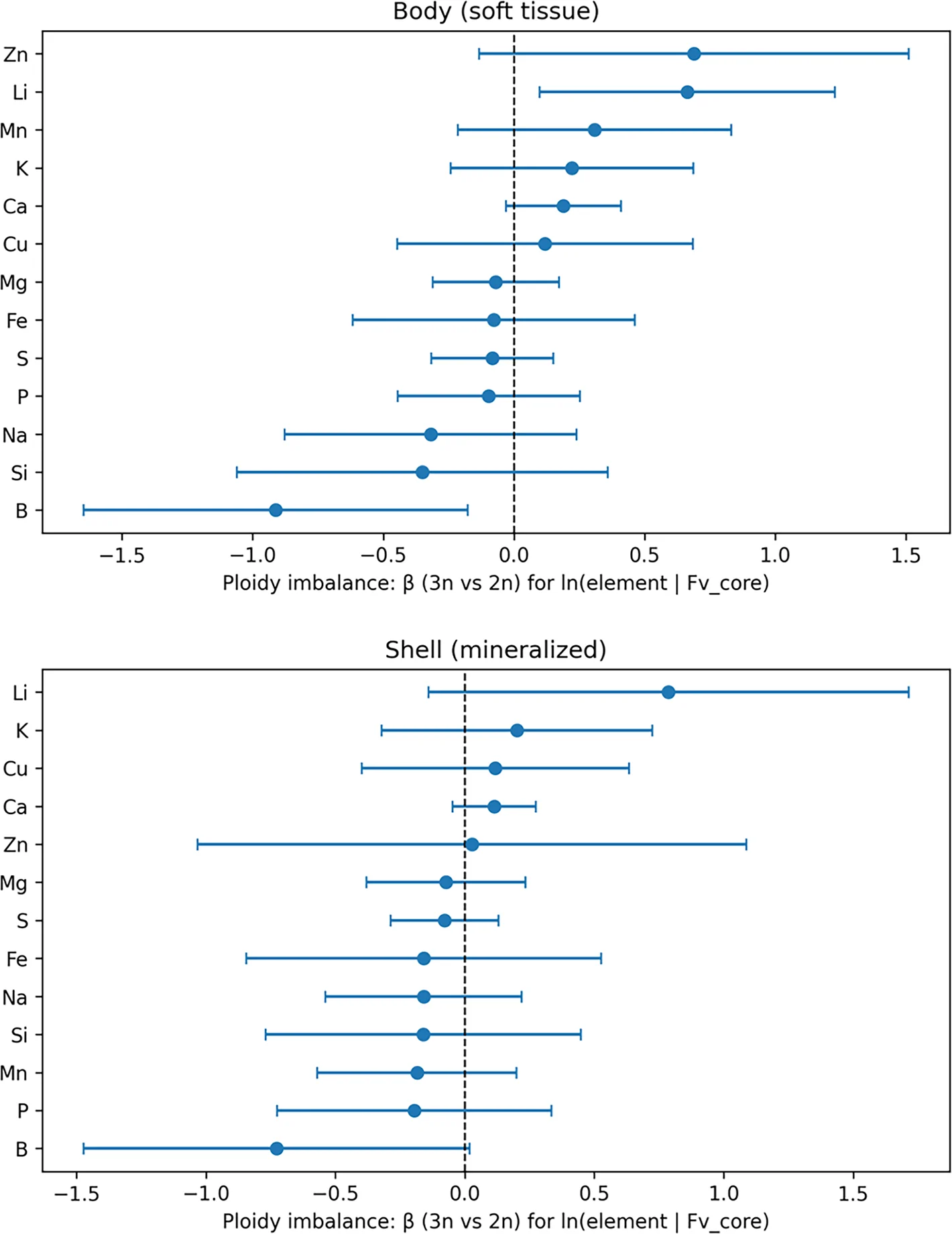
Tissue-specific elemental imbalances associated with ploidy. Ploidy-associated elemental imbalances were quantified using additive log-ratios anchored to the measured elemental pool (ln(element | Fv)), where Fv is the sum of reliably measured elements. Panels show results for metabolically active soft tissue (top) and mineralized shell (bottom). Points indicate estimated ploidy effects (*β*, 3n vs. 2n) from linear models fit separately for each element within tissue, controlling for lake and developmental stage; error bars show 95% confidence intervals. The vertical dashed line at zero represents the diploid (2n) reference state. Values less than zero indicate elements that comprise a smaller fraction of the measured elemental pool in triploids (3n) relative to diploids, whereas values greater than zero indicate elements that comprise a larger fraction of the measured elemental pool in triploids. Confidence intervals that do not overlap zero indicate statistically detectable ploidy-associated imbalance in relative elemental allocation. Similar comparison based on absolute concentration is displayed in supplementary material, figure S1.

In metabolically active soft tissue, ploidy-associated imbalances were generally modest (figure 2, top). By contrast, mineralized shell showed broader directional departures from the diploid reference state (figure 2, bottom), suggesting that persistent ploidy-associated allocation differences may accumulate more clearly in mineralized structures. Elements with confidence intervals not overlapping zero indicate statistically detectable ploidy-associated shifts in their proportional contribution to the measured elemental pool, conditional on Fv and after accounting for lake and developmental stage.

To evaluate whether the observed ploidy signal depended on treating body and shell samples as separate observations, we repeated the analysis using a dataset collapsed to one multielemental observation per individual. The effect of ploidy remained significant (box 1; Wilks's *λ* = 0.759, *F* = 2.095, *p* = 0.022), indicating that the association between ploidy and ionomic composition was robust to the paired body–shell sampling structure.

Because samples originated from multiple isofemale lineages nested within two lakes, we evaluated the robustness of the ploidy signal to lineage structure using a permutation-based sensitivity analysis in which permutations were restricted within lineage. The ploidy-associated multielemental signal remained significant under this more conservative treatment (box 1; PERMANOVA, *F* = 1.64, *R*² = 0.0018, *p* = 0.001), indicating that inference was not dependent on unrestricted exchange of observations among lineages. Because only two lakes were sampled, lake was retained as a fixed effect rather than treated as a random effect. We therefore interpret the observed ploidy effect as applying to the sampled lake-derived lineages rather than as a population-level estimate across lakes. However, the persistence of the signal across multiple lineages suggests that the result is not attributable to a single lineage.

Together, these tissue-specific imbalance profiles illustrate how ploidy-associated elemental reorganization can be resolved differently across biological compartments.

## 4. Discussion

Nuclear DNA content, typically via proxies of genome size or ploidy level, is often invoked as a determinant of organismal performance, yet the material pathways linking genome architecture to phenotype remain difficult to resolve, particularly in animals. By analysing elemental composition as a compositional, multielemental phenotype, we show that snails differing in ploidy level harbour distinct organismal chemistry as a consequence of coordinated redistribution of elements rather than through changes in isolated elemental demands. These effects are specific to body part and element, revealing how nuclear DNA content variation propagates through biological compartments with distinct regulatory and integration timescales.

### (a) Body part defines the chemical backdrop for ploidy effects

Body part overwhelmingly structured ionomic composition (figure 1), confirming that metabolically active soft tissue and mineralized shell occupy distinct chemical regimes. This partition reflects fundamental differences in turnover rate, regulatory control and developmental integration [14,33], suggesting that nuclear DNA content effects act on a chemically compartmentalized organism rather than on a homogeneous pool.

### (b) Ploidy is associated with coordinated multielement redistribution

Although weaker than body part effects, ploidy exerted a statistically detectable multivariate influence on elemental allocation (table 1). Importantly, this effect was expressed as redistribution across multiple elements rather than as strong shifts in any single element. These results extend phosphorus-centred expectations by suggesting that ploidy-associated effects may propagate across broader multielemental organization [9]. Instead, ploidy elevation appears to reorganize the relative partitioning of elements within the measured pool. Similar element-specific rather than phosphorus-dominated shifts have been reported in diploid versus autotetraploid plants, where genome duplication altered Fe, Ca and Zn allocation without consistent increases in bulk %P [18].

Previous work in *P. antipodarum* showed that ploidy alters performance under phosphorus limitation and can influence P use and excretion [5]. The present analysis addresses a complementary question: whether ploidy reorganizes steady-state elemental allocation across a multielement system. The absence of strong bulk %P differences here does not contradict earlier findings; rather, it indicates that nuclear DNA content effects may be expressed through coordinated redistribution that is not detectable through single-element concentration alone.

While extant polyploidy in *P. antipodarum* was discovered decades ago [25], a recent whole-genome assembly for *P. antipodarum* documents an additional relatively recent and now-diploidizing whole-genome duplication that nevertheless still features substantial duplicate retention [34]. Expansion of gene copy number increases the potential landscape of structural macromolecules, transporters and metal-binding proteins, even when expression is buffered. Such expansion provides a mechanistic basis for subtle shifts in uptake, binding, compartmentalization and deposition probabilities across elements. Under this view, ploidy-associated ionomic differences reflect altered system-level allocation dynamics rather than fixed increases in demand for any specific element.

Because ploidy covaries with nuclear DNA content, gene dosage and reproductive mode in this system [6,25,35], the present study cannot isolate which of these features most directly underlies the observed compositional differences. In particular, the differences in reproductive biology and, thus, evolutionary history and dynamics that characterize diploid (obligately sexual) versus triploid and tetraploid (obligately asexual) *P. antipodarum* [35,36] mean that our inference is restricted to ploidy-associated multielemental organization rather than direct causal effects of DNA amount alone. With this limitation in mind, a productive next step would be to compare and contrast the obligately asexual triploids and tetraploids, allowing us to parse out direct consequences of ploidy elevation from those associated with reproductive mode.

### (c) Body part-specific expression of ploidy effects

In both tissues, ploidy-associated imbalances were heterogeneous across elements, indicating selective reorganization of elemental fractions rather than uniform scaling of the ionome (figure 2). The direction and magnitude of these shifts differed among elements and between body compartments, demonstrating that ploidy-associated multielemental reorganization is distributed across the ionome rather than concentrated in a single elemental pool. Soft tissues are subject to active regulation of uptake, storage and turnover, which can buffer steady-state composition despite underlying genomic differences [24,37]. By contrast, shells integrate elemental deposition over extended developmental timescales and are largely decoupled from short-term metabolic regulation [14]. Even small, persistent differences in transport or incorporation rates can therefore accumulate into detectable compositional shifts.

Elements with no established biochemical role in animals, notably lithium and boron, showed some of the clearest ploidy-associated signals. Because these ions interact primarily through weak substitution and non-specific binding [15,22], their redistribution may reflect indirect incorporation, substitution or transport processes associated with broader changes in cellular and mineral organization. This interpretation reinforces the view that ploidy reshapes elemental organization indirectly, through changes in binding landscapes and transport probabilities rather than through selection on simple stoichiometric requirements.

### (d) Implications

Our analysis prioritizes tractability over chemical completeness. We did not quantify bulk constituents such as C, H, O and N, nor elements that are difficult to measure reliably with ICP-based methods (e.g. chloride or ultra-trace metals). The filling value therefore represents the measured elemental pool rather than total organismal chemistry. By expressing composition as log-ratios anchored to this pool, inference is explicitly conditioned on observed chemistry while unmeasured components remain an unspecified remainder [19,20]. Taken together, these results suggest that nuclear DNA content variation leaves a coherent multielemental signature in organismal chemistry, even when single-element effects are weak. Ionomic composition therefore provides a sensitive phenotype for detecting the material consequences of ploidy elevation. By focusing on relative allocation within a measured elemental pool, compositional analysis reveals how genome architecture propagates through rates of uptake, retention and deposition to shape tissue-level chemical organization. More broadly, this approach offers a new way to investigate ploidy and genome size: not only through gene content or performance traits, but through system-level patterns of elemental allocation that integrate genomic, physiological and environmental influences.

## Supplementary Material



## Data Availability

All data are shared as part of the supplementary material [38].

## Declaration of AI use

During preparation and revision of this article, the authors used ChatGPT (OpenAI) to assist with language editing, text compression, organizational restructuring, figure caption refinement, reviewer-response organization and limited coding support. AI-assisted tools were not used for data generation, statistical analysis, compositional calculations, figure production or scientific interpretation. All experimental design, analyses, equations, modelling decisions, interpretations and final writing were reviewed, verified and approved by the authors, who take full responsibility for the published content.
