# Supplementary material for: Ploidy is associated with multielemental reorganization across soft and mineralized body compartments
Source: Biol Lett. 2026 Oct 14;22(10):20260316. doi: 10.1098/rsbl.2026.0316 (PMC13682566; doi:10.1098/rsbl.2026.0316)
Supplement: Supplementary file 1 [file rsbl.2026.0316.zip › rsbl.2026.0316/rsbl20260316_si_001.docx]

**Electronic Supplement for:**

**Title:** Ploidy is associated with multielemental reorganization across soft and mineralized body compartments.

**Authors**; ^1^*Punidan D. Jeyasingh, ^2^Marissa Roseman, ^2^Josh Bliss, ^1^Yetkin Ipek, ^2^Maurine Neiman.

**Affiliations:** ^1^Department of Biology, Oklahoma State University, Stillwater, OK 74078. U.S.A. ^2^Department of Biology, University of Iowa, Iowa City, IA 52242. U.S.A.

*correspondence: [puni.jeyasingh@okstate.edu](mailto:puni.jeyasingh@okstate.edu)

**Running title:** Ploidy effects on ionome

**ESM 1. Supplementary Methods**

*Study system and sampling design*

Lab-reared isofemale lineages of snails were established from field-collected individuals sampled from two South Island, New Zealand lakes. These lakes, Mapourika and Selfe, harbor genetically distinct populations of asexual triploids derived from sympatric sexual diploid conspecifics (Dybdahl and Lively 1995, Paczesniak et al. 2013) and were expected to produce a fairly even mix of both diploid and triploid individuals (Neiman et al. 2011, Paczesniak et al. 2013). We established three replicates from different lineages for each combination of lake of origin, ploidy, and ontogenetic stage. Lineages were housed individually under standard laboratory conditions and fed dried *Spirulina* and calcium supplements in excess.

Sampling ontogenetic stage

We sampled the first ontogenetic stage immediately after juvenile snails emerged from their ovoviviparous mothers, before the juvenile’s shells began to calcify. At this stage, snails are too small to accurately sex, but we used only female snails for the second and third ontogenetic stage because polyploid (asexual) *P. antipodarum* lineages consist almost entirely of females (Neiman et al. 2012) and ionomes can differ drastically between males and females of the same species (Goos et al. 2017). When the remaining snails in the lineage reached 2 mm in length from shell tip to aperture, we sampled the second ontogenetic stage. Upon reproduction signaling sexual maturity, we sampled the third ontogenetic stage. Sexual (diploid) females were housed with males from their lineage after the second ontogenetic stage to allow for fertilization.  To reach the tissue volume necessary in each sample for ion detection levels, we pooled ten individuals for the first ontogenetic stage, five individuals for the second ontogenetic stage, and used a single individual for the third ontogenetic stage.

Ploidy determination

F1 offspring of each isofemale lineage were sexed under the microscope. If the male:female ratios were roughly 50/50, corresponding with predicted sex ratios for diploid sexuals, the lineage was considered as such (Krist et al., 2014). If ploidy could not be determined by sex ratios, we used flow cytometry to compare sample DNA content with standards of known ploidy. For each lineage in question, an individual was dissected, snap-frozen in liquid nitrogen and prepared for flow cytometry following Krist et al. (2014). Flow cytometry was performed on the Becton Dickinson LSR at the Carver College of Medicine’s Flow Cytometry facility. Ploidy was determined by analyzing peak fluorescence values of stained DNA using FloJo software (following, e.g., Neiman et al. 2011).

Specimen Preparation

We fasted adult female snails to reduce sample contamination from undigested food. We recorded the wet mass of each individual, then dissected snails into body and shell components. We separated body tissue and shell fragment into individual microcentrifuge tubes, which were snap-frozen in liquid nitrogen, then stored at -80℃. We ultimately used 124 snails from lake Mapourika (62 body, 62 shell samples) and 84 snails from Selfe n = 84 (42 body, 42 shell). Snails belonged to two ploidy levels: 2n n = 132 (66 body, 66 shell); 3n n = 76 (38 body, 38 shell). For each individual, metabolically active soft tissue (body) and mineralized shell were processed separately (n = 208 tissue samples total; n = 104 body, n = 104 shell). Ploidy (2n vs 3n) and tissue type (body vs shell) were treated as focal biological factors impacting the ionome.

*Sample processing and elemental analysis*

Snail body tissue and shells were processed separately for elemental analysis. Soft tissues and shells were dissected, dried to constant mass, and digested in trace-metal–grade nitric acid (HNO₃) following established ionomic protocols. Digestions were conducted for a minimum of 72 h to ensure complete dissolution of organic material and mineral phases, after which samples were diluted to a known final volume using ultrapure (Type I) water.

Elemental concentrations were quantified using inductively coupled plasma–optical emission spectroscopy (ICP-OES; iCAP 7400, Thermo Scientific, Waltham, MA). A broad multielement panel was measured, including major ions and trace elements relevant to physiological regulation and biomineralization (Al, As, B, Ba, Ca, Cd, Co, Cr, Cu, Fe, K, Li, Mg, Mn, Mo, Na, Ni, P, Pb, S, Se, Si, Sr, V, Zn). Multielement external calibration standards (CPI International, Santa Rosa, CA) were used to calibrate the instrument across the expected concentration ranges, and an inline Yttrium internal standard (Peak Performance Inorganic Y Standard, CPI International) was used to correct for instrument drift and matrix effects.

Procedural blanks were prepared by digesting acid without tissue material and processed alongside samples to account for background concentrations. Elements that were inconsistently detected across samples were retained in the raw dataset but excluded from multivariate analyses where appropriate, as described below.

Elemental concentrations are reported in solution units. Sample mass was unavailable or heterogeneous for a subset of samples, precluding consistent normalization to dry mass. Accordingly, subsequent analyses were designed to quantify relative elemental allocation using log-ratio transformations anchored to the measured elemental pool, rather than absolute elemental content (Greenacre 2021).

*Filling value and denominator definition*

For each sample, we defined a filling value (Fv) as the sum of measured elemental concentrations across a core set of elements that were consistently detected across samples. The core panel comprised 13 elements with ≤5% missing values (B, Ca, Cu, Fe, K, Li, Mg, Mn, Na, P, S, Si, Zn). This core filling value represents the size of the reliably observed elemental pool for each sample. Importantly, the filling value does not assume that all organismal chemistry is measured. Instead, it explicitly conditions inference on the measured chemical pool and treats unmeasured chemistry as an unknown remainder. Negative elemental concentrations were replaced with element-specific limits of detection (LOD), defined as the minimum positive value observed for each element, prior to log-ratio transformation.

*Statistical framework*

To test whether metabolically active soft tissue and mineralized shell occupy distinct multielemental regimes, we analyzed elemental composition using log-ratio methods that respect compositional constraints. Elemental data are inherently compositional because each observation represents relative parts of a constrained whole. Analyses based on raw concentrations can therefore generate spurious correlations when denominator variation is ignored.

To test whether ploidy reorganizes elemental allocation, we computed a version of additive log-ratios (ALRs) for all measured elements (Jeyasingh et al. 2026), using the core filling value as the explicit denominator. For each element $i$, the ALR was defined as:

$$\text{ALR}_{i}=\ln\left( \frac{x_{i}}{F_{v}} \right)$$

where $x_{i}$is the measured concentration of element $i$and $F_{v}$is the core filling value for that sample.

This transformation expresses the relative allocation of each element conditional on the total measured elemental pool and is invariant to unknown multiplicative scaling factors such as sample mass or dilution.

To test whether ploidy effects differ between metabolically active soft tissue and inert shells, we fit multivariate models including body part, ploidy, their interaction, lake, and developmental stage. We then conducted body part-specific analyses to evaluate ploidy effects within soft tissue and shell separately while controlling for environmental and ontogenetic context.

Statistical analyses were conducted using multivariate and univariate models applied to ALR-transformed data. Multivariate differences in ionomic composition were evaluated using MANOVA, with Wilks’ lambda used for inference. We diagnosed ploidy-associated elemental imbalances using ALRs anchored to the measured elemental pool, analogous to imbalance analyses previously used to quantify deviation from ancestral ionomic states (Jeyasingh et al. 2023). Diploid (2n) *P. antipodarum* represent the ancestral form (e.g., Neiman et al. 2011) and were thus treated as the reference state, such that ploidy effects represent log-ratio deviations in elemental allocation in triploids relative to diploids.

**ESM 2.** **Supplementary Data.** Mass-specific elemental concentrations and metadata for *Potamopyrgus antipodarum* ionome analyses. Merged dataset containing sample metadata (lake, ploidy, developmental stage, lineage, body part) and elemental concentrations for all measured elements. Concentrations are provided in solution units (µg/L).

**ESM 3. Supplementary Figure 1.**

**Fig. S1. Body part-specific differences in absolute elemental concentrations associated with ploidy.** Panels show ratios of mean absolute elemental concentrations in triploid (3n) relative to diploid (2n) *Potamopyrgus antipodarum* for metabolically active soft tissue and mineralized shell. Points indicate estimated concentration ratios (3n/2n) for individual elements; error bars show 95% confidence intervals derived from linear models fit separately for each element within body part, controlling for lake and developmental stage. The vertical dashed line at 1 represents no concentration difference between ploidy levels. Values greater than 1 indicate higher absolute elemental concentrations in triploids, whereas values less than 1 indicate lower absolute elemental concentrations in triploids. Because elemental concentrations are compositionally constrained and cannot vary independently, formal statistical inference was conducted on additive log-ratios (Figure 2; Table 1).
